# Facilitating deep learning through preprocessing of optical coherence tomography images

**DOI:** 10.1186/s12886-023-02916-2

**Published:** 2023-04-17

**Authors:** Anfei Li, James P Winebrake, Kyle Kovacs

**Affiliations:** 1 Department of Ophthalmology, New York Presbyterian Hospital, 1305 York Ave 11th floor, New York, NY 10021 USA; 2 Department of Ophthalmology, Weill Cornell Medicine, 1305 York Ave 11th floor, New York, NY 10021 USA

**Keywords:** Deep learning, Machine learning, Preprocessing, Optical coherence tomography

## Abstract

**Background:**

While deep learning has delivered promising results in the field of ophthalmology, the hurdle to complete a deep learning study is high. In this study, we aim to facilitate small scale model trainings by exploring the role of preprocessing to reduce computational burden and accelerate learning.

**Methods:**

A small subset of a previously published dataset containing optical coherence tomography images of choroidal neovascularization, drusen, diabetic macula edema, and normal macula was modified using Fourier transformation and bandpass filter, producing high frequency images, original images, and low frequency images. Each set of images was trained with the same model, and their performances were compared.

**Results:**

Compared to that with the original image dataset, the model trained with the high frequency image dataset achieved an improved final performance and reached maximum performance much earlier (in fewer epochs). The model trained with low frequency images did not achieve a meaningful performance.

**Conclusion:**

Appropriate preprocessing of training images can accelerate the training process and can potentially facilitate modeling using artificial intelligence when limited by sample size or computational power.

**Supplementary Information:**

The online version contains supplementary material available at 10.1186/s12886-023-02916-2.

## Background

Increased use of artificial intelligence has revolutionized the way humans interact with computers [1]. Deep learning allows computer algorithms the freedom to select features without extensive human input, thereby transcending historical roles. In ophthalmology, deep learning has been applied across almost all subspecialties including retina, glaucoma, neuro-ophthalmology, and oculoplastics [2–6]. Specifically, retinal optical coherence tomography images have been tested extensively via deep learning to identify diagnoses, clinical features, and anatomical retinal layer segmentation [7, 8]. Similar feats have been accomplished by dermatologists, using OCT images for detection of skin pathologies and layer segmentation [9, 10]. While the potential success of unsupervised learning and deep learning is promising, they are not without shortcomings. Although unsupervised learning with deep learning provides a significant advantage over prior supervised machine learning approaches by allowing for identification of previously unexpected features for disease classification [11], an enormous amount of resources are required for such analysis. Performance barriers include the need for large training datasets, sophisticated models, and computational power availability. Thus, major artificial intelligence studies, particularly image-based deep learning studies in ophthalmology, have been mostly constrained to large academic centers or multi-institutional groups, limiting the field's participation in advancement of artificial intelligence research. From a practical perspective, many retinal diseases do not meet the threshold for deep learning-based analysis due to the relatively limited case numbers. With this limitation in mind, the goal of the present study was to devise a way in which reasonable deep learning training can be achieved with lower resources, particularly sample size and model complexity. We re-trained a subset of a publicly available dataset of retinal optical coherence tomography (OCT) images using a smaller training sample size and a simplified model after preprocessing the original images with Fourier transformation and a bandpass filter [12]. In particular, images produced by high-bandpass filter that contained the finer details of the original image as opposed to the gross shape/form led to improved performance compared to using the original images, especially when the training sample size was small. This result suggests that appropriate image preprocessing through refinement of features may allow the extension of deep learning into areas of rare diseases in which sample availability has thus far prohibitively excluded its application.

## Methods

### Image selection and preprocessing

**Fig. 1:**
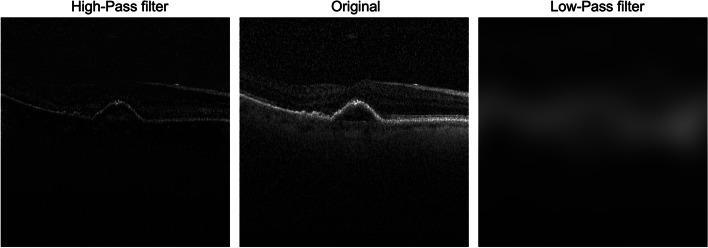
Representative optical coherence tomography images of high-frequency image (left), original image (middle), and low-frequency image (right). The same threshold was used for both high-pass and low-pass filter, high frequency and low frequency images together represents all features of the original image

Previously published images by Kermany et. al were obtained from a publicly available Mendeley database (10.17632/rscbjbr9sj.3) [12]. While the original study used multiple images from the same subject (one eye or both eyes), only the first representative image (image -1 as denoted by original authors) was used for each subject so that each subject only contributed a single image to the training. As such, image -1 did not always contain the most representative slice for the corresponding pathology (or at all in some cases). However, this selection method minimized human subjectivity and was more likely to produce a smaller training set that best resembled the composition of the original training set. A total of 797 choroidal neovascularization (CNV), 768 diabetic macular edema (DME), 716 drusen, and 3437 normal images were used for the analysis. Original images were cropped with a python script evenly from all sides to 500x500 pixel images containing the fovea, further reducing training burden. Fast Fourier transformation was applied to all images using fft2() function in numpy.fft python package. After spatial signals were transformed into frequencies, frequency threshold at which high and lower frequencies produced equal spatial signals was determined empirically for 10 random images. The average of the 10 thresholds were used as population threshold. For each image transformed to frequency domain, all frequencies lower than the threshold was inversely transformed back to spatial signals to produce the low-frequency image, and all frequencies higher than the threshold was inversely transformed back to spatial signals to produce the high-frequency image. ifft() function from numpy.fft package was used for inverse transformation. In doing so, a corresponding low frequency image and a corresponding high image were produced for any given original image, each carrying roughly half of the original features/spatial signals from the original image (Fig. 1).

### Deep learning and training model

Keras package (version 2.3.1) from python was used for deep learning. Publicly available pre-trained VGG16, VGG19, ResNet50, and Xception models with additional dropout layer (0.5) and dense layer (softmax activation) were used for training. VGG16 model consisted of 138M parameters and a depth of 16, VGG19 model consisted of 144M parameters and depth of 19, ResNet50 model consisted of 26M parameters and a depth of 107 (with skip connections), and Xception model consisted of 23M parameters and depth of 81 (with separable convolutional layers). Further details of the models can be found on the Keras official site (https://keras.io/api/applications/). The models were fit using weighted training to account for an imbalanced training set, and fitting hyperparameters were set to a learning rate of 0.005, decay of 1x10^-6^, momentum of 0.9, batch size of 5, and epochs of 25. Each training dataset – original images, high frequency images, and low frequency images – was trained individually with training, validation, and testing splits at a 6:2:2 ratio. For each model, Area-Under-the-Curve (AUC) was calculated for each pathology (against the rest) using the OneVsRestClassifier() function from sklearn.metrics python package as a measure of performance. Additional testing with the traditional training, validation, and testing splits at an 8:1:1 ratio was done using the VGG16 base-model to study the effect of larger training sample size. GPUtil module on python was used to monitor graphics processing unit (GPU) and memory load during preprocessing and model training.

## Results

**Fig. 2:**
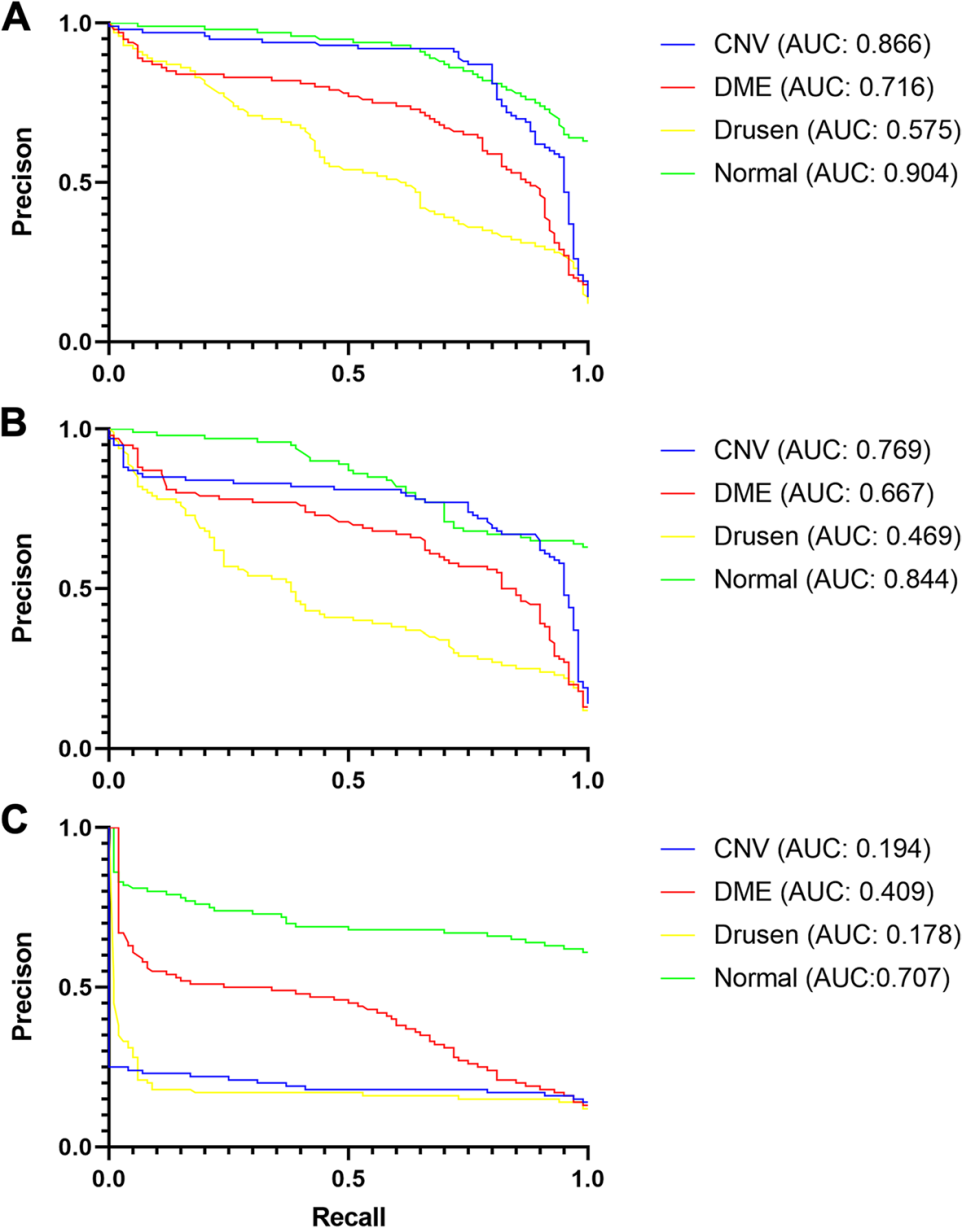
Precision-Recall (PR) Curves for each image set and Area Under the Curve (AUC) for each pathology vs. the rest. **A** PR curve for high-frequency images, Blue: CNV AUC = 0.866±0.037, Red: DME AUC = 0.716±0.048, Yellow: Drusen AUC = 0.575±0.054, Green: Normal AUC = 0.904±0.017. **B** PR curve for original images, Blue: CNV AUC = 0.769±0.045, Red: DME AUC = 0.667±0.049, Yellow: Drusen AUC = 0.469±0.052, Green: Normal AUC = 0.844±0.022. **C** PR curve for low-frequency images, Blue: CNV AUC = 0.194±0.030, Red: DME AUC = 0.409±0.046, Yellow: Drusen AUC = 0.178±0.030, Green: Normal AUC = 0.707±0.029

Using the VGG16-base model and smaller training sample size (60% of dataset), the original images achieved a final average AUC of 0.687, and the high frequency images achieved a higher final average AUC of 0.765. In contrast, the low frequency images had a final average AUC of 0.372 (Fig. 2 and Table 1). Confusion matrix showed that while the high frequency image-based model identified all pathologies with relatively high accuracy, comparable accuracies were achieved by the original image-based model only in the setting of high false positive rates (Supplemental Table 1). Furthermore, the use of high-frequency images led to faster training compared to using the original images, even though the discrepancy in final accuracies was not as large. While high frequency image training reached 70% validation accuracy within 5 epochs, original image training did not reach 70% until the 23^rd^ epoch (or 68% until the 19^th^ epoch; Supplemental Figure 1). Final testing accuracy was tested after 25 epochs of training as both training and validation accuracies did not improve further after around 25 epochs. At 35 epochs and beyond, validation accuracies began to deteriorate for high-frequency images and original images likely due to overtraining, and this trend was largely similar regardless of training sample size or base models.

**Table 1:**
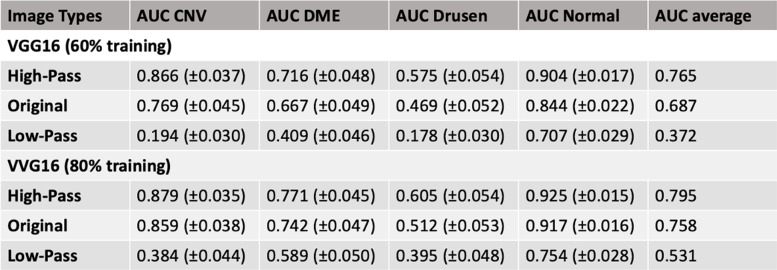
Area-under-the-curve and 95% confidence interval for each pathology vs rest

GPU load and memory use were tracked and showed that GPU load varied between 94-99% and memory load remained at 89% during training for all image types (Supplemental Figure 2A). CPU load varied between 15-25% during the training process (data not shown). For preprocessing of original images into the high- and low-frequency images, there was minimal GPU and memory usage (<1% and 10%, respectively; Supplemental Figure 2B) and 16-27% CPU load.

When VGG16-base model was retrained using a larger training set (80% of the total dataset), normal and high-frequency images achieved similar average AUCs of 0.758 and 0.795 (Table 1). Additionally, each image set was trained with different models including VGG19, ResNet50, and Xception using the smaller training sample size (60% training set). Average AUCs for VGG19 model trained with normal, high-frequency, and low-frequency images were 0.680, 0.778, and 0.360, respectively. Average AUCs for ResNet50 model trained with normal, high-frequency, and low frequency images were 0.763, 0.804, and 0.482, respectively. Average AUCs for Xception model trained with normal, high-frequency, and low frequency images were 0.789, 0.808, and 0.425, respectively (Table 2).

**Table 2:**
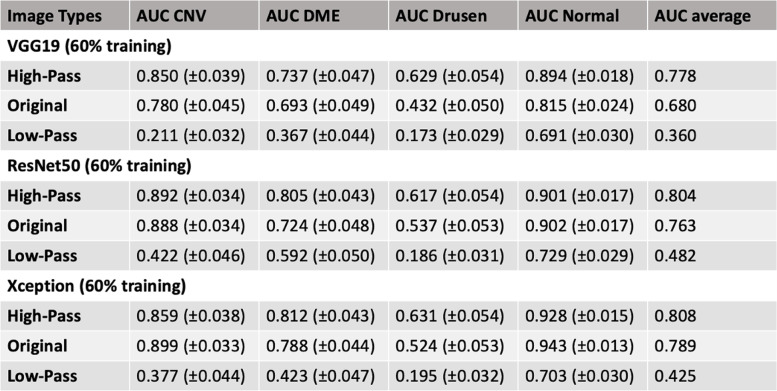
Area-under-the-curve and 95% confidence interval for each pathology vs rest

## Discussion

In their original work, Kermany et. al. elegantly demonstrated the power of deep learning with application to automated diagnosis of common retinal conditions based on OCT images[12]. In this study, we demonstrated that through Fourier transformation and frequency-based filtering, learning can be directed towards a relatively focused set of features while still preserving the benefit of flexible unsupervised learning within the set. In theory, this represents a middle ground between machine learning and deep learning. Specifically, our results demonstrated that when distinguishing normal retina from drusen, DME, and CNV, high frequency images likely contain more useful information or features than low frequency images. Using the VGG16 model, an improved performance was achieved when trained with high frequency images compared with the original images. In fact, the model trained with high frequency images also achieved and plateaued at its final accuracy much earlier compared to the model trained with normal images, although the performance did begin to converge after 15 epochs of training. The accelerated training and improved final performance may be due to offloading of the nonspecific low frequency features during the training process. That said, with enough sample size and model complexity, the original images should eventually achieve similar or even better performance, as it contains all the features. Meanwhile, low frequency features, which illustrate the general contour of the overall retina, expectedly struggled with classification accuracy.

Specifically, when the training sample size was limited and model architecture was simplified (VGG16 vs Inception V3 used by the original article), the normal images achieved a relatively limited average AUC of 0.687, indicating that good performance largely depended on the size of the training set and the complexity of the model architecture. However, our results demonstrate that by extracting high frequency details or features using Fourier transformation, the final performance of the model can be enhanced, achieving an average AUC of 0.765, without increasing training sample size or model complexity. The use of Fourier transformation allowed us to change images from the spatial domain into the frequency domain where the high frequency signals corresponded to fine details within the spatial domain (such as each retinal layer) and low frequency signals corresponded to the gross shape and form of the entire image in the spatial domain (such as overall retinal contour). By splitting the overall details of the images in half in the frequency domain (i.e., bandpass filter), high-frequency images containing fine details of retinal layers and low-frequency images containing gross retinal contour can be produced by transforming frequencies back to the spatial domain through inverse transformation. Given that subretinal neovascular formation in CNV, intraretinal fluid in DME, and sub-retinal pigmented epithelium (sub-RPE) deposits in drusen are all expected to significantly alter retinal layers (and with DME also altering gross retinal contour), high frequency images that capture these key layers expectedly performed well. On the other hand, as details of most retinal layers were missing from the low frequency images, they did not perform as well. Notably, the model trained with low frequency images classified almost all images as DME. While this may be purely coincidental, it is also possible that the model is biased towards gross retinal contour change seen in DME, which is well captured by the low frequency images.

In addition to improved final performance, high frequency images also exhibited accelerated training speed which may allow for truncation of training duration. While complex measurements and calculations of computational efficiency is beyond the scope of this project, if training is truncated to when maximum validation performance is achieved, truncated training with high-frequency images may reduce the duration of the training by a factor of 1.33 compared to the full training with the original image; consequently, reducing the net GPU and memory use by factors of 2.5 and 2.3, respectively. While cropping the images to smaller size also helped accelerate training and reduce computational burden, identical cropping was applied to all three groups, and therefore will not affect the relative difference in training and performance among the groups.

It should be noted that improved performance of VGG16 model trained with high frequency images does not immediately imply that all deep learning using OCT images should be focused on high frequency images. First of all, as delineated above, the benefit of high frequency images demonstrated in our manuscript is, in theory, due to intrinsic changes to the retinal layers in the pathologies contained within the dataset. An alternative pathology that exhibits more gross retinal contour change, central serous retinopathy for example, may benefit from low frequency imaging or a different preprocessing method all together. Secondly, as demonstrated by additional modeling with different training set size or different models, the benefit of this preprocessing method is most apparent when there is limitation to training sample size or model depth. As shown by VGG16 modeling using a larger training set (80% of dataset), while high frequency images continued to perform well, original images achieved a similar final performance, challenging the superiority of the high frequency images. Similarly, when VGG19, ResNet50, and Xception-based models were trained with the three data sets, results differed based on the base model. While high frequency images led to good performance in all models, a benefit over use of the original images was only detected in the VGG19 model. Conversely, ResNet50 and Xception models trained with original images showed similar performance to those trained with high frequency images. Compared to VGG16 or VGG19 architectures, the ResNet50 and Xception architectures are significantly deeper with 107 and 81 layers, respectively. Additionally, RestNet50 and Xception architectures involve more complex designs, namingly skip connections that do not exist in the VGG models. These results demonstrate that the benefit of high frequency images only becomes apparent when the training set size and/or model depth are constrained. In other words, by preprocessing the image set, it is possible to approach the performance level of a bigger data set or a more complex model while using a smaller data set or a simpler and shallower model architecture.

It is not easy to ascertain how preprocessed images (or inputs) interact differently with each layer of the deep learning model. However, we speculate that the ability for the high frequency images to improve performance in the setting of low sample size and shallower model may be due to increased efficiency for features to be optimally weighted at each hidden layer. The increased proportion of useful features in high-frequency images after the filtering process may have helped to mitigate the disadvantages of reduced instances of useful features (due to smaller sample size) or decreased rounds of weight adjustments (due to shallower architecture). While adding additional depth to VGG models may not seem challenging, development of progressively deeper and more complex architectures may be quickly outgrowing the capabilities of computational hardware [13]. Similarly, rare or novel diseases will pose challenges to acquiring large sample sizes for training, especially in an era when previously larger unified diagnoses are being subdivided further to discrete entities. Therefore, there will be situation in which preprocessing through filtering – bandpass filter being one of them – may enable meaningful model training when we cannot acquire sufficient samples. In such cases, our approach to preprocess/filter the images prior to training may serve as a facilitation step to overcome limitations posed by sample size, model architecture, or both.

It is important to acknowledge that preprocessing is not necessarily an unexplored area in deep learning. However, prior work on preprocessing of images for deep learning has been focused largely on augmenting the dataset to improve generalizability [14, 15]. For example, the sample size of the training dataset – and consequently number of unique features/orientations – can be increased by multiple folds through rotations, translations, shifting, etc. The ultimate goal of augmentation was to construct a more comprehensive dataset that increased the robustness of the final model. Consequently, this has also led to an increase in the overall computational burden. In contrast, our approach using Fourier transformation and bandpass filter attempted to construct a more efficient dataset by trimming away the relatively irrelevant features. By choosing the appropriate method to filter features, as we have demonstrated, better final performance can be achieved with the same sample size and model. It should be noted that preprocessing for augmentation and filtering are not mutually exclusive as both methods can be used in series when preprocessing images. In fact, they may be additive or even synergistic as both are intended to present the model with as many relevant features as possible during training.

The present study is limited to a particular image preprocessing method on a specific imaging modality, focused on a specific region of interest (ROI). We are not implying that Fourier transformation will improve performance for all trainings, or that high bandpass filter is always superior to low bandpass filter. Instead, as a proof of concept, we want to demonstrate that the Fourier transformation and high bandpass filter is an appropriate preprocessing method to accelerate and improve training for distinguishing drusen, DME, and CNV on OCT images. Conversely, low bandpass filters may be appropriate for training of entities with more global tissue disruption (such as a retinal detachment detection using fundus photos), as the overall regional difference may be more telling than fine details. In this study, intensities of the high frequency and low frequency images were equalized through bandpass thresholding for academic comparison purposes. In reality, bandpass filter can be set to any threshold to achieve maximum training performance, tailored to the specific image type. Similarly, the degree of benefit achieved by such filtering may depend on sample selection, which is not further investigated in the present study. As the model trained in the original article has largely achieved performance ceiling, we downsized the training sample size to better demonstrate performance improvement after preprocessing. Different downsizing approaches, therefore, may bias the resulting training sets differently and yield varied degrees of benefit.

For our future endeavors, we look to investigate other filter-based preprocessing methods that may contribute to improved training with deep learning models, with an attempt to truly apply the methods to rarer diseases. Other processing methods, ranging from segmentation to contrast enhancement, may all be useful when applied to the right images for the appropriate trainings. For example, red-green filter may be an appropriate filtering method for preprocessing of wide-field fundus photos for identification of various peripheral retinal lesions. Speckle noise removal may be another possible preprocessing method that could enhance performance of models trained with OCT images. Literatures from both ophthalmology and dermatology have found success in removing speckle noises using unsupervised learning (K-means) or deep learning [16, 17]. In theory, OCT images without noises should result in improved modeling and performance, although running additional unsupervised learning or deep learning just for image preprocessing may not be an worthwhile approach.

Finally, we must emphasize that the Fourier transformation approach introduced here or filter-based preprocessing in general are not meant to be an upgrade to the current state-of-the-art deep learning. Rather, it offers another option in situations when an aspect of the ideal deep learning set up is limited: whether it be computational hardware, sample size, model complexity, or time. In an ideal world, original images from a large dataset should be used to train an appropriately deep model in order to achieve the most robust performance. By preprocessing the data, we actively hinder what may be the essence of deep learning: freedom to choose useful features with minimal human interference. However, with a small amount of guidance, it has the potential to reduce the minimal sample size or model complexity needed to achieve meaningful training, opening up applications for scholars without access to large computational cores or databases. While small scale training using this approach does not replace large scale studies by large centers, it can potentially bridge between human-guided machine learning and machine-driven deep learning to facilitate influx of ideas into the fields of artificial intelligence and ophthalmology. Just as importantly, it offers an approach in which deep learning can be implemented to relatively rare diseases or small subpopulations in which it is unrealistic to train full-scale deep learning models based on large datasets. Similar benefit also extends to early implementation of deep learning in small preliminary datasets (e.g., novel imaging modalities, status-post novel treatments) to guide future research directions.

## Conclusion

With increased availability of large datasets including images, artificial intelligence is beginning to play a larger role in analytical works as well as practical clinical functions. However, the prevalence of artificial intelligence research can be limited by relatively stringent requirement for large sample sizes, sophisticated modeling, and high-end computational hardware. In this study, we demonstrated that training of deep learning models can be facilitated by utilizing Fourier transformation-based preprocessing, achieving improved final performance when sample size and model complexity were suboptimal. Our approach is not meant to surpass or compete with the current state-of-the-art deep learning modeling using large datasets and deep network architectures. Additionally, the specific Fourier transformation-based approach highlighted in this manuscript is unlikely to benefit other imaging modalities or diseases to similar a degree. However, as we begin to bring deep learning into areas of relatively rare diseases, the generalized approach for refining features during preprocessing, in addition to augmentation, may open opportunities for successful modeling in areas never imagined before.

## Supplementary Information

**Additional file 1:** **Supplemental Table 1.** Confusion metrix for each model.

**Additional file 2:** **Supplemental Figure 1.**Training and validation accuracies using original image (green), high frequencyimage (blue), and low frequency (red). Training curves are plotted every 5steps and smoothed by a factor of 0.6 for visualizationpurposes. **Supplemental Figure 2.** GPU and memory usage. A. GPU load and memory usage during training for original image (green), high frequency image (blue), and low frequency (red). GPU load varied between 94-99% and memory usage remained at 89% throughout the training session. B. GPU load and memory usage for preprocessing images to high- and low-frequency images.

## Data Availability

The data that support the findings of this study are openly available in Mendeley at https://doi.org/10.17632/rscbjbr9sj.3

## References

[1] Shinde PP, Shah S. A Review of Machine Learning and Deep Learning Applications. Proc - 2018 4th Int Conf Comput Commun Control Autom ICCUBEA 2018. 2018. 10.1109/ICCUBEA.2018.8697857

[2] Armstrong GW, Lorch AC (2020). A(eye): A Review of Current Applications of Artificial Intelligence and Machine Learning in Ophthalmology. Int Ophthalmol Clin..

[3] Balyen L, Peto T (2019). Promising artificial intelligence–machine learning–deep learning algorithms in ophthalmology. Asia-Pacific J Ophthalmol..

[4] Oke I, VanderVeen D (2021). Machine Learning Applications in Pediatric Ophthalmology. Semin Ophthalmol..

[5] Li Z, Qiang W, Chen H, Pei M, Yu X, Wang L, Li Z, Xie W, Wu X, Jiang J, Wu G (2022). Artificial intelligence to detect malignant eyelid tumors from photographic images. NPJ Digit Med.

[6] Milea D, Najjar RP, Jiang Z, Ting D, Vasseneix C, Xu X, AghsaeiFard M, Fonseca P, Vanikieti K, Lagrèze WA, La Morgia C, Cheung CY, Hamann S, Chiquet C, Sanda N, Yang H, Mejico LJ, Rougier M-B, Kho R (2020). Artificial Intelligence to Detect Papilledema from Ocular Fundus Photographs. N Engl J Med..

[7] Pekala M, Joshi N, Liu TYA, Bressler NM, DeBuc DC, Burlina P (2019). Deep learning based retinal OCT segmentation. Comput Biol Med.

[8] Schlegl T, Waldstein SM, Bogunovic H, Endstraßer F, Sadeghipour A, Philip AM, Podkowinski D, Gerendas BS, Langs G, Schmidt-Erfurth U (2018). Fully Automated Detection and Quantification of Macular Fluid in OCT Using Deep Learning. Ophthalmology.

[9] Turani Z, Fatemizadeh E, Blumetti T, Daveluy S, Moraes AF, Chen W, Mehregan D, Andersen PE, Nasiriavanaki M (2019). Optical radiomic signatures derived from optical coherence tomography images improve identification of melanoma. Cancer Res.

[10] Lin C-H, Rajabi-Estarabadi A, May J, Pang Y, Dai Y, Avanaki K. Epidermal Thickness Measurement on Skin OCT Using Time-Efficient Deep Learning with Graph Search.; 2022. 10.1117/12.2613041

[11] Ongsulee P. Artificial intelligence, machine learning and deep learning. Int Conf ICT Knowl Eng. 2018:1-6. 10.1109/ICTKE.2017.8259629

[12] Kermany DS, Goldbaum M, Cai W, Valentim CCS, Liang H, Baxter SL, McKeown A, Yang G, Wu X, Yan F, Dong J, Prasadha MK, Pei J, Ting M, Zhu J, Li C, Hewett S, Dong J, Ziyar I (2018). Identifying Medical Diagnoses and Treatable Diseases by Image-Based Deep Learning. Cell.

[13] Thompson NC, Greenewald K, Lee K, Manso GF. The Computational Limits of Deep Learning. arXiv. 2020. http://arxiv.org/abs/2007.05558.

[14] Khosla C, Saini BS (2020). Enhancing Performance of Deep Learning Models with different Data Augmentation Techniques: A Survey. Proc Int Conf Intell Eng Manag ICIEM.

[15] Khalifa NE, Loey M, Mirjalili S (2022). A comprehensive survey of recent trends in deep learning for digital images augmentation. Artif Intell Rev..

[16] Shi F, Cai N, Gu Y, Hu D, Ma Y, Chen Y, Chen X (2019). DeSpecNet: a CNN-based method for speckle reduction in retinal optical coherence tomography images. Phys Med Biol.

[17] Eybposh MH, Turani Z, Mehregan D, Nasiriavanaki M (2018). Cluster-based filtering framework for speckle reduction in OCT images. Biomed Opt Express.

