# Supplementary figures and images for: Facilitating deep learning through preprocessing of optical coherence tomography images
Source: BMC Ophthalmol. 2023 Apr 17;23:158. doi: 10.1186/s12886-023-02916-2 (PMC10108538; doi:10.1186/s12886-023-02916-2)

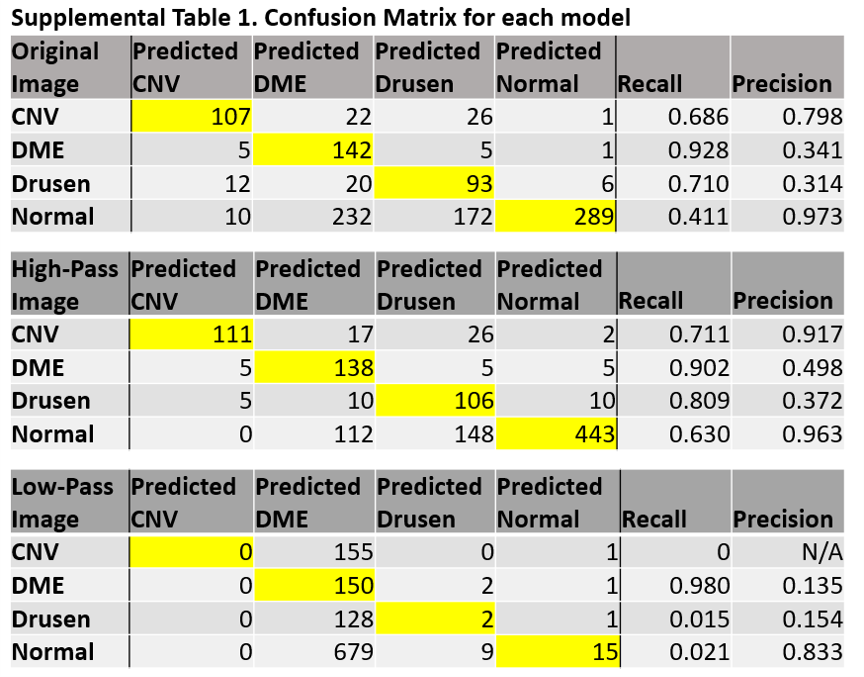

Supplement: Supplementary file 1 — Additional file 1: Supplemental Table 1. Confusion metrix for each model. [file 12886_2023_2916_MOESM1_ESM.png]
