# Supplementary material for: Facilitating deep learning through preprocessing of optical coherence tomography images
Source: BMC Ophthalmol. 2023 Apr 17;23:158. doi: 10.1186/s12886-023-02916-2 (PMC10108538; doi:10.1186/s12886-023-02916-2)
Supplement: Supplementary file 2 — Additional file 2: Supplemental Figure 1.Training and validation accuracies using original image (green), high frequencyimage (blue), and low frequency (red). Training curves are plotted every 5steps and smoothed by a factor of 0.6 for visualizationpurposes. Supplemental Figure 2. GPU and memory usage. A. GPU load and memory usage during training for original image (green), high frequency image (blue), and low frequency (red). GPU load varied between 94-99% and memory usage remained at 89% throughout the training session. B. GPU load and memory usage for preprocessing images to high- and low-frequency images. [file 12886_2023_2916_MOESM2_ESM.docx]

﻿
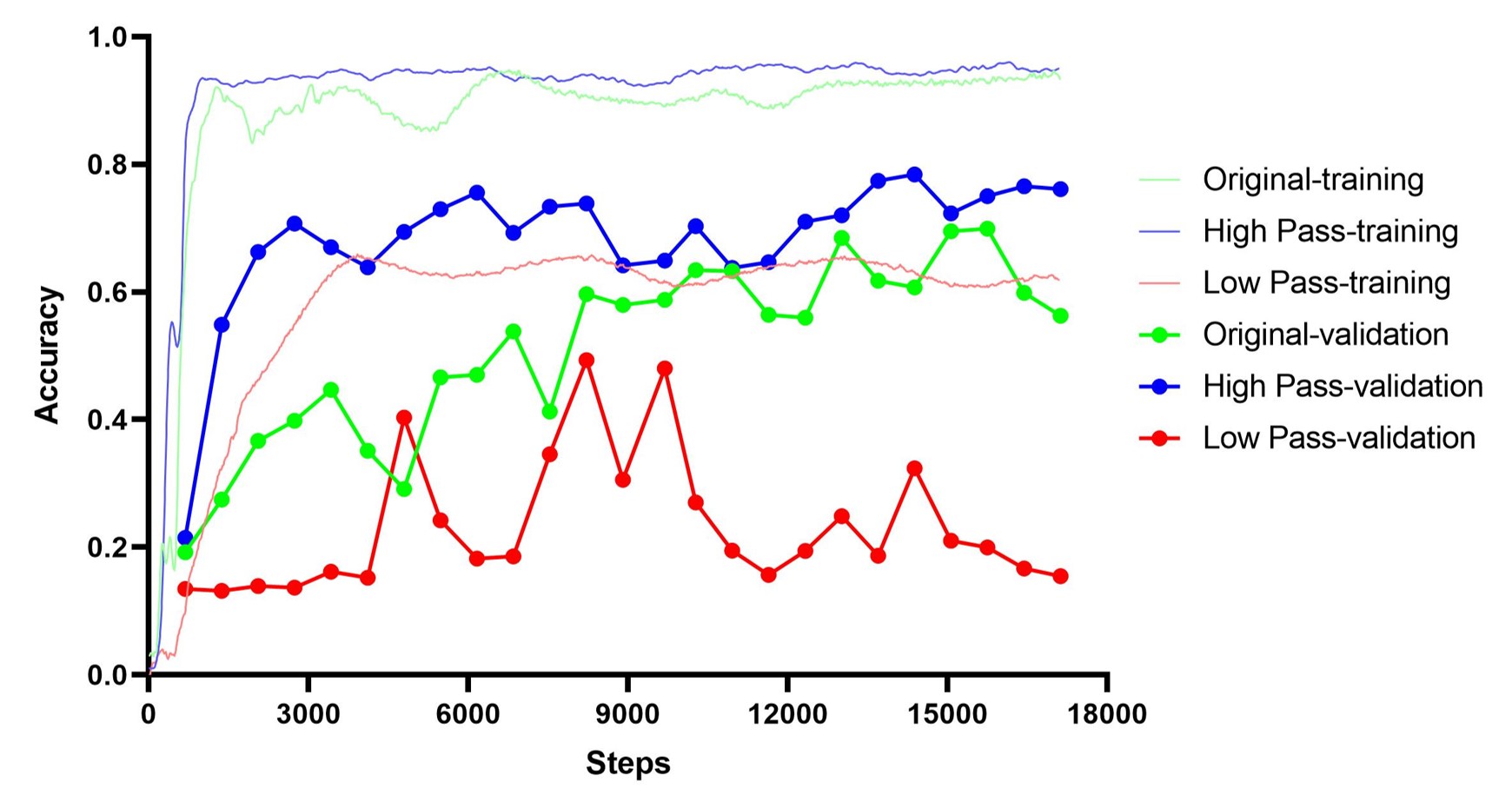


**Supplemental Figure 1.** Training and validation accuracies using original image (green), high frequency image (blue), and low frequency (red). Training curves are plotted every 5 steps and smoothed by a factor of 0.6 for visualization purposes.


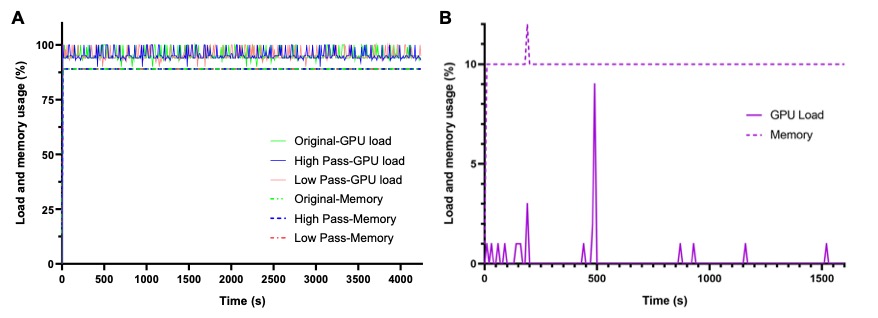


**Supplemental Figure 2.** GPU and memory usage. **A.** GPU load and memory usage during training for original image (green), high frequency image (blue), and low frequency (red). GPU load varied between 94-99% and memory usage remained at 89% throughout the training session. **B.** GPU load and memory usage for preprocessing images to high- and low-frequency images.
